# How to engage the right brain hemisphere in aphasics without even singing: evidence for two paths of speech recovery

**DOI:** 10.3389/fnhum.2013.00035

**Published:** 2013-02-27

**Authors:** Benjamin Stahl, Ilona Henseler, Robert Turner, Stefan Geyer, Sonja A. Kotz

**Affiliations:** ^1^Department of Neurophysics, Max Planck Institute for Human Cognitive and Brain SciencesLeipzig, Germany; ^2^Department of Neurology, Max Planck Institute for Human Cognitive and Brain SciencesLeipzig, Germany; ^3^Department of Neuropsychology, Max Planck Institute for Human Cognitive and Brain SciencesLeipzig, Germany

**Keywords:** left-hemispheric stroke, non-fluent aphasia, melodic intonation therapy, singing, rhythmic speech, formulaic language, left perilesional brain regions, right corticostriatal brain areas

## Abstract

There is an ongoing debate as to whether singing helps left-hemispheric stroke patients recover from non-fluent aphasia through stimulation of the right hemisphere. According to recent work, it may not be singing itself that aids speech production in non-fluent aphasic patients, but rhythm and lyric type. However, the long-term effects of melody and rhythm on speech recovery are largely unknown. In the current experiment, we tested 15 patients with chronic non-fluent aphasia who underwent either singing therapy, rhythmic therapy, or standard speech therapy. The experiment controlled for phonatory quality, vocal frequency variability, pitch accuracy, syllable duration, phonetic complexity and other influences, such as the acoustic setting and learning effects induced by the testing itself. The results provide the first evidence that singing and rhythmic speech may be similarly effective in the treatment of non-fluent aphasia. This finding may challenge the view that singing causes a transfer of language function from the left to the right hemisphere. Instead, both singing and rhythmic therapy patients made good progress in the production of common, formulaic phrases—known to be supported by right corticostriatal brain areas. This progress occurred at an early stage of both therapies and was stable over time. Conversely, patients receiving standard therapy made less progress in the production of formulaic phrases. They did, however, improve their production of non-formulaic speech, in contrast to singing and rhythmic therapy patients, who did not. In light of these results, it may be worth considering the combined use of standard therapy and the training of formulaic phrases, whether sung or rhythmically spoken. Standard therapy may engage, in particular, left perilesional brain regions, while training of formulaic phrases may open new ways of tapping into right-hemisphere language resources—even without singing.

## Introduction

Left-hemispheric stroke patients often suffer a profound loss of spontaneous speech, known as non-fluent aphasia. Yet, many patients are still able to *sing* entire pieces of text fluently (Mills, 1904; Gerstmann, 1964; Yamadori et al., 1977). Unsurprisingly, this finding has drawn much scientific attention in the last few decades. Attention has been mainly focused on two research questions: from a cross-sectional view, one may ask whether it is singing itself that enables aphasic patients to produce text; from a longitudinal view, one may ask whether one could use singing to aid speech recovery. These questions have inspired a growing scientific debate and a number of singing therapies (Keith and Aronson, 1975; Van Eeckhout et al., 1997; Jungblut, 2009), among them a rehabilitation program known as melodic intonation therapy (Albert et al., 1973; Sparks et al., 1974; Helm-Estabrooks et al., 1989). This therapy is based on three main components: singing, rhythmic speech, and common phrases. According to the inventors of the therapy, singing is supposed to stimulate the intact right hemisphere, which then assumes the function of damaged left-hemisphere speech areas.

Recent cross-sectional evidence, however, points in a different direction. An experiment with 17 non-fluent aphasic patients suggests that singing may not facilitate speech production over and above rhythmic speech (Stahl et al., 2011). The rates of correct syllable production were found to be similar when patients were singing and speaking rhythmically. Furthermore, the results indicate that speech production in patients with extensive left-sided basal ganglia lesions may critically depend on external rhythmic cues—such as percussion beats. Patients with larger basal ganglia lesions produced more syllables correctly when they were singing or speaking with rhythmic accompaniment, as compared to when speaking with arrhythmic accompaniment. This effect was not observed in patients with smaller basal ganglia lesions, where changes in rhythmicity did not seem to affect articulatory quality. Finally, the results also confirmed that common, formulaic phrases (e.g., “How are you?”) may have a strong impact on speech production in non-fluent aphasic patients. Formulaic phrases yielded higher rates of correct syllable production than non-formulaic phrases—whether they were sung or rhythmically spoken.

The role of formulaic phrases in singing therapies is critical, as the right hemisphere supports more than just features related to singing (Riecker et al., 2000; Callan et al., 2006; Özdemir et al., 2006). There is growing evidence that the right hemisphere also supports the processing of formulaic language. Several studies suggest that the production of formulaic speech engages *right* corticostriatal areas (Speedie et al., 1993; Van Lancker Sidtis et al., 2003; Van Lancker Sidtis and Postman, 2006; Sidtis et al., 2009). Thus, the ability to produce formulaic expressions is often preserved in left-hemispheric stroke patients (Lum and Ellis, 1994). Conversely, the recovery of non-formulaic, propositional speech may involve, in particular, *left* perilesional regions (Cao et al., 1999; Warburton et al., 1999; Kessler et al., 2000; Rosen et al., 2000; Zahn et al., 2004; Meinzer et al., 2008). This finding is consistent with the observation that the suppression of right-hemisphere brain activity in left-sided stroke patients may facilitate recovery of propositional language (Martin et al., 2009; You et al., 2011). In sum, formulaic and propositional speech may be lateralized differently in the brain (Van Lancker Sidtis, 2004).

Right corticostriatal processing of formulaic language may shed new light on imaging studies that have reported right-hemispheric changes in aphasic patients after singing therapy. In multiple-case reports, aphasic patients were singing formulaic phrases over a period of several weeks (Schlaug et al., 2008, 2009). At the end of this training, the patients' speech had improved. Moreover, functional magnetic resonance imaging (fMRI) and diffusion tensor imaging (DTI) suggested functional changes in the right hemisphere (Schlaug et al., 2008) and structural changes in the right arcuate fasciculus (Schlaug et al., 2009). In fact, these changes may not necessarily relate to singing, as they could just as well arise from the use of formulaic language. Furthermore, right corticostriatal processing of formulaic language may help to better understand the results of a frequently discussed positron emission tomography (PET) study with seven aphasic patients (Belin et al., 1996). All of these patients had previously undergone singing therapy. Unexpectedly, PET revealed increased *left* prefrontal activation in the patients when they were singing simple, concrete words. Several methodological reasons may account for this finding—such as lyric type. It should be noted that the patients in this study were producing non-formulaic utterances, engaging primarily left perilesional brain regions. Hence, neurophysiological correlates in the context of singing may be strongly influenced by whether or not formulaic language is used.

So far, longitudinal evidence for the efficacy of singing in speech recovery is sparse, and a closer look at the studies that do exist reveals some experimental problems. Only two case reports made use of a control condition: one study controlled for singing in an experienced singer (Wilson et al., 2006) and another study controlled for singing, but not for rhythmic left-hand tapping, in two patients (Schlaug et al., 2008). Consequently, the results from these reports may be confounded by musical training and influences related to rhythm. Nonetheless, some longitudinal work provides evidence for the efficacy of rhythmic pacing in speech recovery (Rubow et al., 1982; Pilon et al., 1998; Brendel and Ziegler, 2008). The results of these studies suggest that speech recovery may be modulated by auditory, visual, or tactile rhythmic cues. It may therefore be critical that melodic intonation therapy includes rhythmic hand tapping. Tactile stimulation, such as tapping of the left hand, may affect speech production by engaging sensorimotor networks in the right hemisphere (Gentilucci and Dalla Volta, 2008). In other words, rhythmic pacing may have a strong impact on speech recovery in aphasic patients.

Until now, it remains unclear whether or not singing conveys any therapeutic advantage over rhythmic speech. Moreover, there is no evidence as to how well patients can switch between singing and rhythmic speech if their training is focused on either singing or rhythmic speech. Finally, it is unclear whether possible progress in the production of formulaic phrases extends to the production of non-formulaic, propositional speech. With the current experiment, we aimed to address these questions. In a longitudinal design, we investigated the relative clinical effects of melody and rhythm on the recovery of formulaic and non-formulaic speech in non-fluent aphasic patients.

## Materials and methods

### Participants

The present multicenter study was conducted at five rehabilitation centers located in Berlin, Germany, between 2009 and 2012. Fifteen stroke patients were included in the study. Table 1 provides an overview of the patients' individual case histories. Patients were German native-speakers, right-handed, and aged 40–72 years (mean age: 56 years; standard deviation: 10 years). Except for three patients with previous infarctions (patients LS, OK, PH), none of the patients had a pre-morbid history of neurological or psychiatric impairments, nor did any of the patients suffer from dementia. None of the patients had hearing problems or complained of impaired hearing. To restrict influences related to spontaneous recovery, all patients were at least 6 months post-infarction at the time of testing, suggesting a chronic post-stroke stage. Eight independent speech-language pathologists tested the patients within 1 month prior to the study, using a German standard aphasia test battery (*Aachen Aphasia Test*, Huber et al., 1984). Specified test scores are given in Table 2.

**Table 1:** **Patient histories**.

**Patient**	**Gender**	**Age**	**Months since**	**Number of**	**Aetiology**	**Left-sided**	**Sensorimotor**	**Handedness**
		**(years)**	**last infarction**	**infarcts**		**lesions include**	**deficits**	
IK	M	61	9	1	Left MCA ischemia	FT cortex, insula, BG	Paresis (R)	R
LS	F	53	36	2	Left MCA ischemia	FT cortex, insula, BG, thalamus	Paresis (R), hypesthesia (R)	R
OK	M	62	12	2	Left BG hemorrhage	Insula, BG	Paresis (R), hypalgesia (R)	R
PL	M	49	6	1	Left MCA ischemia	FT cortex, insula, BG, thalamus	Paresis (R), hypesthesia (R)	R
PR	F	58	156	1	Left MCA ischemia	FPT cortex, insula, BG, thalamus	Paresis (R)	R
AS	F	65	8	1	Left MCA ischemia	FT cortex, insula, thalamus	None	R
DO	M	47	14	1	Left MCA ischemia	FT cortex, insula, BG, thalamus	Paresis (R), hypesthesia (R)	R
GB	M	71	23	1	Left MCA ischemia	FT cortex, insula, BG	Paresis (R)	R
HG	F	40	10	1	Left MCA hemorrhage	FT cortex, insula	Paresis (R)	R
PH	M	72	6	2	Left MCA ischemia	FPT cortex, insula	Paresis (R)	R
CM	M	47	33	1	Left MCA ischemia	FT cortex, insula, BG	Paresis (R)	R
HK	F	52	10	1	Left MCA ischemia	FT cortex, insula, BG	Paresis (R), hypalgesia (R)	R
HP	F	68	6	1	Left BG hemorrhage	Insula, BG, thalamus	Paresis (R), hypesthesia (R)	R
JD	M	53	16	1	Left MCA ischemia	FT cortex, insula, BG	None	R
TJ	F	45	7	1	Left MCA ischemia	FT cortex, insula, BG, thalamus	Paresis (R)	R

*Patients are sorted by treatment group (from* top *to* bottom*): singing therapy (patients IK, LS, OK, PL, PR), rhythmic therapy (patients AS, DO, GB, HG, PH), and standard therapy (patients CM, HK, HP, JD, TJ).*

M, male; F, female; R, right; MCA, middle cerebral artery; FT, fronto-temporal; FPT, fronto-parieto-temporal; BG, basal ganglia.

**Table 2:** **Language assessment**.

**Patient**	**Token test**	**Comprehension**	**Naming**	**Repetition**	***Diagnosis***
IK	16/50	90/120	57/120	100/150	Broca's aphasia; moderate AOS
LS	31/50	57/120	0/120	24/150	Global aphasia; moderate-severe AOS
OK	26/50	74/120	19/120	37/150	Global aphasia; mild-moderate AOS
PL	14/50	99/120	60/120	77/150	Broca's aphasia; severe AOS; mild dysarthria
PR	9/50	112/120	75/120	102/150	Broca's aphasia; moderate AOS
AS	2/50	120/120	99/120	122/150	Broca's aphasia; mild-moderate AOS
DO	29/50	58/120	8/120	53/150	Global aphasia; moderate AOS
GB	36/50	61/120	2/120	102/150	Global aphasia; mild-moderate AOS
HG	16/50	98/120	58/120	72/150	Broca's aphasia; severe AOS
PH	37/50	63/120	0/120	8/150	Global aphasia; severe AOS
CM	5/50	102/120	0/120	61/150	Broca's aphasia; moderate-severe AOS
HK	26/50	72/120	0/120	58/150	Global aphasia; mild-moderate AOS
HP	24/50	76/120	5/120	85/150	Global aphasia; mild dysarthria
JD	10/50	115/120	92/120	103/150	Broca's aphasia; moderate AOS
TJ	19/50	72/120	5/120	11/150	Global aphasia; severe AOS
Singing therapy group mean (SD)	19/50 (±9.0)	86/120 (±21.5)	42/120 (±31.3)	68/150 (±35.9)	–
Rhythmic therapy group mean (SD)	24/50 (±14.9)	80/120 (±27.6)	33/120 (±43.7)	71/150 (±44.3)	–
Standard therapy group mean (SD)	17/50 (±9.0)	87/120 (±19.9)	20/120 (±40.1)	64/150 (±34.7)	–

*Scores of the* Aachen Aphasia Test. *Token test: no/mild disorder (0–6); light (7–21); middle (22–40); severe (>40). Comprehension (including words and sentences in both the visual and auditory modality): no/mild disorder (104–120); light (87–103); middle (58–86); severe (1–57). Naming: no/mild disorder (109–120); light (92–108); middle (41–91); severe (1–40). Repetition: no/mild disorder (144–150); light (123–143); middle (75–122); severe (1–74). Severity levels of apraxia of speech and dysarthria are based on the ratings in the patients' clinical files.*

*Patients are sorted by treatment group (from* top *to* bottom*): singing therapy (patients IK, LS, OK, PL, PR), rhythmic therapy (patients AS, DO, GB, HG, PH), and standard therapy (patients CM, HK, HP, JD, TJ).*

AOS, apraxia of speech; SD, standard deviation.

Patients were diagnosed with Broca's aphasia (*n* = 7) or global aphasia with prevailing expressive deficits (*n* = 8). Non-fluent aphasia usually concurs with speech disorders that include difficulties in planning and executing oral, speech-specific movements (apraxia of speech), or coordinating articulatory organs, respiration, and the larynx (dysarthria). To increase diagnostic reliability, concomitant speech disorders in the studied patients had to be diagnosed by at least two experienced speech-language pathologists. Patients were diagnosed with apraxia of speech on the basis of direct observations, which involved inconsistently occurring phonemic or phonetic errors, word initiation difficulties, and visible groping (see Brendel and Ziegler, 2008). Correspondingly, dysarthria was diagnosed in case of consistently occurring phonetic errors. As a result, the diagnosed concomitant speech disorders in the current patient sample involved apraxia of speech (*n* = 14) and dysarthria (*n* = 2).

Patients were eligible for inclusion in the study when the aphasia test results indicated preserved simple comprehension, with comparably limited verbal expression. It should be noted that the patients were considered “non-fluent” based on the typological classifications indicated by the aphasia test (global or Broca's aphasia). Moreover, the speech-language pathologists diagnosed non-fluent aphasia as a prevailing disorder in all of the patients. All patients had undergone speech therapy, which did not comprise singing or explicit rhythmic speech. None of the patients displayed any specific musical training or experience in singing. The sample may therefore be considered as exemplary in a clinical context.

CT and MRI scans, as well as relevant medical reports, were obtained for all patients. A neurologist with special expertise in neuroradiology (I.H.) re-analyzed all CT and MRI scans to determine the homogeneity of the current sample in terms of lesion site. The results of these analyses are shown in Table 1. All patients suffered from ischemia in the left middle cerebral artery, except for three patients with left hemisphere hemorrhages (patients HG, HP, OK). The right hemisphere was intact in all patients. The study was approved by the Ethical Committee at University of Leipzig and by the participating clinics in Berlin, and informed consent was obtained from all patients.

### Stimuli

**Figure 1:**
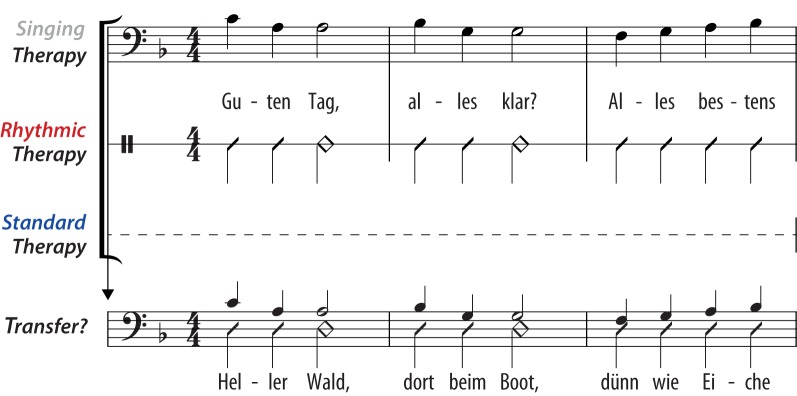
**Schematic overview of the experimental design.** Three types of treatment were applied: singing therapy, rhythmic therapy, or standard therapy. In *singing therapy*, patients underwent training of common, formulaic lyrics by singing them to a well-known melody (“Hello, everything alright? Everything's fine…”). In *rhythmic therapy*, patients were trained using the same lyrics, but rhythmically spoken with natural prosody. In *standard therapy*, patients attended speech therapy that did not include singing, rhythmic speech, or training with formulaic phrases. In each treatment group, the production of formulaic lyrics was assessed at different stages of the therapy. Finally, it was explored whether the patients showed a *training transfer* to the production of unknown, non-formulaic lyrics that were not part of any treatment (“Bright forest, there at the boat, thin like oak…”).

The experimental design focused on singing, rhythmic speech, and lyric type. A schematic overview of the design is given in Figure 1. Three types of treatment were applied: singing therapy, rhythmic therapy, or standard therapy. In singing therapy, patients underwent intense training of formulaic lyrics by singing them to a well-known melody. In rhythmic therapy, patients were trained using the same formulaic lyrics, but rhythmically spoken with natural prosody. In standard therapy, patients attended speech therapy that did not include singing, rhythmic speech, or training with formulaic phrases. In each treatment group, the production of formulaic lyrics was assessed at different stages of the therapy. Finally, it was explored whether the patients showed a training transfer to the production of unknown, non-formulaic lyrics that were not part of any treatment. Rhythmic therapy served as the control condition for singing therapy, whereas non-formulaic lyrics provided the control for formulaic lyrics. All stimuli were piloted in a previous, published study, which we will, henceforth, refer to as “pilot work” (Stahl et al., 2011).

A highly familiar song was chosen (*Hänschen klein*), based on pilot work with 35 healthy participants. The song proved to be highly familiar, irrespective of the participants' age. In pilot work with 17 aphasics, familiarity with the melody did not constrain the patients' sung production of lyrics that differed from the original ones. This result suggests that familiarity with a melody does not interfere with lyric production in aphasic patients. Hence, the use of a familiar melody in the current experiment appears to be an appropriate choice. The melody mainly consists of thirds, while not exceeding the range of a fifth. Melodic intonation therapy is largely based on thirds, therefore the chosen melody is suitable as it exhibits similar properties.

Formulaic lyrics were composed of stereotyped phrases (“Hello, everything alright? Everything's fine … ”). In pilot work, eight clinical linguists were asked to judge over 100 common phrases, and classified half of them as being “formulaic.” Fifteen of these phrases were chosen and combined to form a sequence that could be found in typical “small talk.” The phrases are highly relevant for communication in everyday life, ranging from salutations and farewells to well-being and food. The sequence of phrases showed high word transition frequencies, indicating high co-occurrences between adjacent words. Notably, the sequence of formulaic phrases was based both on the linguists' judgments, and on word transition frequencies that may be viewed as a psycholinguistic marker for overlearnedness.

In a next step, we developed lyrics to assess the production of non-formulaic, propositional speech before and after therapy. However, formulaic phrases and non-formulaic speech are often difficult to distinguish, because even remote expressions may be or may become formulaic in a given communicative context. Consequently, non-formulaic lyrics had to be largely devoid of stereotyped expressions and common word transitions to meet the requirements of the present study. Non-formulaic lyrics therefore included very unlikely, but syntactically correct phrases, such as might occur in modern poetry (“Bright forest, there at the boat, thin like oak…”). Low word transition frequencies were used as a psycholinguistic marker to avoid high co-occurrences of words. As a result, non-formulaic lyrics showed significantly lower word transition frequencies than formulaic lyrics [*t*_(66)_ = 2.23, *p* = 0.029].

One may imagine that singing therapy favors sung production of phrases, whereas rhythmic therapy favors spoken production of phrases. For this reason, all lyrics were tested both sung and rhythmically spoken, whether they were part of the treatment or not. It is important to note that formulaic and non-formulaic lyrics did not differ in: word frequency [*t*_(68)_ < 0.01, not significant (n.s.)]; word frequency variance [*F*_(34, 34)_ = 1.09, n.s.]; syllable frequency [*t*_(68)_ = 0.45, n.s.]; number of consonants; and syntactic phrase structure. Both lyric types were consistent with the rhythmically required meter in German. The meter is trochaic, meaning that stressed syllables are always followed by unstressed syllables or a short pause. Table 3 provides some characteristics of the lyrics.

**Table 3:** **Characteristics of the lyrics**.

**Feature**	**Formulaic lyrics**	**Non-formulaic lyrics**
Mean word frequency (CI)	110,900 (±58,289)	110,921 (±67,376)
Mean word transition frequency (right neighbor)	4,609	0
Mean syllable frequency (CI)	10,881 (±8096)	13,615 (±11,459)
Number of words	35	35
Number of syllables	49	49
Number of consonants	82	82
Number of syllable onsets with: two consonants; one consonant; vowel only	2; 40; 7	4; 39; 6
Number of ellipsoidal phrases	15	14

*Syllable frequencies have been computed based on the CELEX database (Baayen et al., 1993). Further values were taken from the online database* Wortschatz Leipzig *(University of Leipzig, www.wortschatz.uni-leipzig.de).*

CI, confidence interval.

To assess speech production at different stages of therapy, the patients sang or spoke along to a playback composed of a pre-recorded voice to mimic and a percussion beat. Percussive accompaniments were chosen to control for tempo, as syllable duration may affect speech production (Beukelman and Yorkston, 1977; Laughlin et al., 1979; Hustad et al., 2003). Notably, percussion beats are usually not part of spoken utterances in everyday life. However, the presence or absence of rhythmic accompaniments did not interfere with speech production in four pilot patients. The use of percussion beats may therefore provide an effective control of syllable duration in the present experiment.

Playback voice and percussion beat were mixed in the recording, with both tracks being separately normalized. The sound intensity level of the percussion beat was decreased by 10 dB to make both tracks clearly audible. A male singer performed both the sung and spoken vocal playback parts. The sung playback parts were recorded in two tonal keys (B and F major) to represent the patients' individual vocal range, with a piano sound indicating the initial note. Natural prosody was employed for the spoken playback parts. The playback voice was digitally edited to ensure that each syllable was precisely placed on the beat. For the percussion beat, a wooden metronome sound was used. The first percussion beat in every 4/4 measure was stressed by lowering the percussion frequency and by accentuating its intensity (first beat in every measure: fundamental frequency of 280 Hz, sound intensity level of 80 dB; all remaining beats: fundamental frequency of 420 Hz, sound intensity level of 70 dB). Based on pilot work, a tempo of 100 beats per minute was chosen, with a mean duration of 780 ± 25 ms per syllable. With this tempo, patients produced about half of the syllables correctly, thus indicating a medium difficulty level. Every condition was primed by two measures of percussion beats. Examples of the playbacks can be downloaded at http://www.cbs.mpg.de/~stahl.

### Treatments

The patients were allocated to one of the following treatment groups: singing therapy (patients IK, LS, OK, PL, PR), rhythmic therapy (patients AS, DO, GB, HG, PH), or standard therapy (patients CM, HK, HP, JD, TJ). It should be noted that the patients did *not* receive any other treatment throughout the entire study phase. Given the limited overall sample size, patients were systematically assigned to the different treatments based on the following criteria: clinical diagnosis (Broca's or global aphasia); severity of concomitant apraxia of speech; age; and gender. The purpose of this assignment process was to make the treatment groups as comparable as possible. As a result, each treatment group consisted of two patients with Broca's aphasia, except for three patients with Broca's aphasia in the singing therapy group. Furthermore, the treatment groups were comparable in terms of severity of concomitant apraxia, mean age (57, 59, and 53 years for singing, rhythmic, and standard therapy, respectively), and gender (about half women). Also, Mann-Whitney *U* tests did not yield significant differences between any of the treatment groups in the language assessment scores shown in Table 2 (*z* ≤ 0.94, always n.s.). All patients underwent three 1-h long, weekly training sessions, over a period of 6 weeks. Every session was conducted individually in one rehabilitation center.

The singing therapy was structured into three training levels. Every 2 weeks patients advanced to the next level. This time interval was chosen based on additional pilot work with two patients. After about 2 weeks, patients were able to double the rate of correctly produced syllables, suggesting a distinct progress in treatment. At level one, patients were singing formulaic lyrics, with the experimenter (B.S.) singing along (“Guten Tag, alles klar…”). At level two, the experimenter was singing along just the metrically prominent syllables, thus omitting the unstressed syllables (“Gu—Tag, al—klar…”). The procedure was piloted with five patients, who could produce phrases much better if metrically prominent syllables were sung or spoken along. This may be due to the use of a trochaic meter in German, in which stressed beats often concur with initial word syllables. Hence, metrical cues may have helped the patients to overcome word initiation difficulties. At level three, the patients were singing alone without any help provided by the experimenter. One further aim at level three was to integrate the formulaic phrases in the patients' everyday environment at home. Small cards were labeled with single phrases and attached to objects that could be meaningfully related to each other (e.g., “I am hungry” on the fridge, “Did you sleep well?” on the bedside table). In other words, patients and their relatives were encouraged to use the phrases appropriately in a given everyday context. Also, at this level, the patients' relatives attended the therapy sessions, whenever possible.

Rhythmic therapy was structured in exactly the same way, the only difference being that patients were not singing the lyrics, but rhythmically speaking them. It may be obvious that both singing and rhythmic therapy contain rhythmic elements, simply because rhythm is naturally inherent in singing. However, singing and rhythmic therapy in the present study clearly differed in whether the patients were intentionally singing or not. Moreover, rhythmic left-hand tapping was not allowed in any of the treatment groups, as hand tapping may act as an additional therapeutic element, which would limit the interpretation of the data.

Speech therapy usually involves a number of different elements. For the purpose of standardizing speech therapy, an experienced clinical linguist was asked to compose commonly used elements in the treatment of non-fluent aphasia and apraxia of speech. This composition was supposed to satisfy current clinical standards (Barthel et al., 2008). The most frequent elements applied include: multi-modal stimulation (receptive: categorization, word-picture matching; expressive: repetition, reading aloud, naming, writing); simplifying strategies (“reduced syntax therapy”; Springer et al., 2000); phonetic or phonemic approach (“minimal contrast treatment”; Wambaugh et al., 1996); tactile-kinaesthetic speech-motor treatment (“prompts for restructuring oral and muscular phonetic targets”; Square-Storer and Hayden, 1989); and communicative-pragmatic approach (“promoting aphasics' communicative effectiveness”; Davis and Wilcox, 1985). Five experienced clinical linguists delivered the standard therapy in one rehabilitation center.

### Measurements

The production of *formulaic lyrics*, both sung and rhythmically spoken, was tested before and after 6 weeks of each treatment. To explore gradual training effects, singing and rhythmic therapy involved additional interim measurements after 2 and 4 weeks. Furthermore, singing and rhythmic therapy included follow-up testing of formulaic lyrics 3 months after the end of the treatment. In both groups, interviews with the patients' relatives were conducted to explore how well-formulaic phrases were used at home after therapy. The interviews focused on three questions: the patients' adequate use of formulaic phrases according to communicative contexts; the actual number of trained phrases transferred to everyday life; the degree to which patients depended on external cues during phrase production over the course of the treatment. The production of *non-formulaic lyrics*, both sung and rhythmically spoken, was tested before and after 6 weeks of each treatment.

One may claim that several interim measurements are likely to cause learning effects induced by the testing itself. This especially applies to the testing of formulaic lyrics in standard therapy, as well as to the testing of non-formulaic lyrics in each treatment group. To rule out this issue, standard therapy did not include interim measurements of formulaic lyrics, nor did any of the treatment groups involve interim measurements of non-formulaic lyrics. Furthermore, one may argue that follow-up testing in standard therapy may have been desirable from an experimental point of view. However, follow-up testing in this group would have required the patients to not attend any kind of conventional speech therapy during a period of 3 months after the end of the experiment. Otherwise, it may have been difficult to ensure that the follow-up results actually arose from the experimental treatment. Since it poses ethical problems to exclude severely affected patients from treatment for such a long time, standard therapy did not include follow-up testing in the current experiment. In case of singing and rhythmic therapy, none of the patients received repetitive training of formulaic speech during a period of 3 months after the end of the experiment. Consequently, the follow-up results in both of these groups are likely to reflect experimental progress.

Each measurement took place in one session with pauses in between, according to the patients' individual needs. To avoid carryover effects, modalities (sung, spoken), and lyric types (formulaic, non-formulaic) were presented in separate blocks: formulaic lyrics spoken; formulaic lyrics sung; non-formulaic lyrics spoken; non-formulaic lyrics sung. Patients produced the stimuli in each block four times. Spoken stimuli were always presented first, as an association of melody and lyrics could have interfered with spoken lyric production.

It was assessed whether learning effects occurred during the measurements, separately for each time of testing. This is important because each testing session alone may have induced long-term learning effects, irrespective of the treatment applied. Note that this control analysis did not focus on progress in speech production over a period of weeks, but on possible progress occurring during each testing session. Given the limited number of trials per condition, non-parametric rank correlation analyses (Kendall's τ_*b*_) between the rate of correct syllables and the corresponding trial number were performed separately for each time of testing and lyric type. The results suggested learning effects in two patients, always occurring during one testing session (formulaic lyrics: patients IK and TJ; τ_*b*_ = 0.69, 0.96, *p* = 0.018 and *p* < 0.001; non-formulaic lyrics: patients IK and TJ; τ_*b*_ = 0.76, 0.89, *p* = 0.009, 0.003). However, none of the patients showed a deviant result pattern in how their speech production improved over a period of weeks in each treatment group. In other words, it seems rather unlikely that any testing alone may account for long-term learning effects in the patients.

For all measurements, patients were seated in front of two loudspeakers at a distance of 75 cm. Patients listened to the vocal playback to sing or speak along with, while being provided with separate sheets of text for each lyric type. It should be noted that lip-reading was not possible because it may affect the performance of non-fluent aphasic patients (Fridriksson et al., 2012). Again, rhythmic hand tapping was not allowed as it may have facilitated speech production by engaging the sensorimotor system. The acoustic setting was conceived to resemble choral singing, with auditory feedback originating from the singer's own voice, as well as from surrounding sound sources. In pilot work with five healthy participants, the playback intensity was chosen to be approximately balanced with the singer's perceived own vocal loudness. Auditory feedback was not given via earphones to preserve natural vocal self-monitoring. Utterances were recorded using a head microphone (C520 Vocal Condenser Microphone, AKG Acoustics, Vienna, Austria) and a digital recording device (M-Audio Microtrack II, Avid Technology, Burlington, MA).

### Data analysis

Two speech-language pathology students independently rated the articulatory quality of the produced utterances based on the digital sound files, with two raters for each patient. The speech-language pathology students were not aware of the expected outcome of the experiment. Articulatory quality was denoted as the percentage of correct syllables in each condition. Syllables were chosen over words as the critical unit to account for the fact that, in apractic patients, errors often occur at the syllable level (Aichert and Ziegler, 2004). A total number of 33,840 syllables were rated. The analyses focused on the segmental sound structure at both the phonemic and the phonetic level. The first two syllables in each condition were discarded from the analyses to control for onset difficulties. Correct syllables were scored with one point (formulaic lyrics: 48% of syllables; non-formulaic lyrics: 13%). Half points were given in two conditions: phonemic or phonetic errors occurring in one or more consonants per syllable, but not in the vowel—and vice versa (formulaic lyrics: 27% of syllables; non-formulaic lyrics: 27%). No points were allocated when errors occurred in both the vowel and in one or more of the consonants within a syllable (formulaic lyrics: 21%; non-formulaic lyrics: 56%). Further errors were classified as syllable substitutions for part of a different word (formulaic lyrics: 2%; non-formulaic lyrics: 1%) or omissions (formulaic lyrics: 2%; non-formulaic lyrics: 3%). This scoring procedure has proven efficient in previous studies (Racette et al., 2006; Stahl et al., 2011). Inter-rater reliabilities for articulatory quality in each patient resulted in correlations ranging from 0.97 to 1.00, with an overall inter-rater reliability across patients of 0.99, *p*(218) < 0.001.

Pitch accuracy was assessed for each sung syllable. It is noteworthy that pitch accuracy did not significantly differ between the lyric types [mean pitch accuracy of formulaic lyrics: 78%; non-formulaic lyrics: 75%; *t*_(14)_ = 1.33, n.s.], nor did it significantly differ between any of the treatment groups (mean pitch accuracy in patients undergoing singing therapy: 77%; rhythmic therapy: 80%; standard therapy: 64%; for each group comparison: Mann–Whitney *U* test, *z* ≤ 0.84, always n.s.). Moreover, the pitch accuracy scores before therapy failed to predict subsequent changes in speech production after 6 weeks of therapy in any of the treatment groups, as revealed by non-parametric correlation analyses (Kendall's τ_*b*_), with an overall correlation across treatment groups of 0.34, n.s.

Average scores of articulatory quality were computed, composed of two raters' judgments for each condition and patient. Based on these scores, a repeated measures analysis of covariance (ANCOVA) was performed, including the factors time (before treatment, after 6 weeks of treatment), lyrics (formulaic, non-formulaic), and modality (sung, spoken), with treatment group as between-subject factor (singing therapy, rhythmic therapy, standard therapy). To control for pre-treatment differences between subjects, baseline scores were included as a covariate (Overall and Doyle, 1994; Van Breukelen, 2006). Pre-treatment performances in the different conditions, including both modalities (sung, spoken) and lyric types (formulaic, non-formulaic), were averaged for each patient to compute individual baseline scores. For additional *post-hoc* frequency analyses the software *Praat* was used (Boersma and Weenink, 2012). The requirements for the repeated measures ANCOVA with small samples were met: according to Shapiro–Wilk tests, the data were normally distributed, and the standard deviations in each condition did not differ much in size, ranging from 16 to 22. An alpha level of 0.05 was applied.

## Results

**Figure 2:**
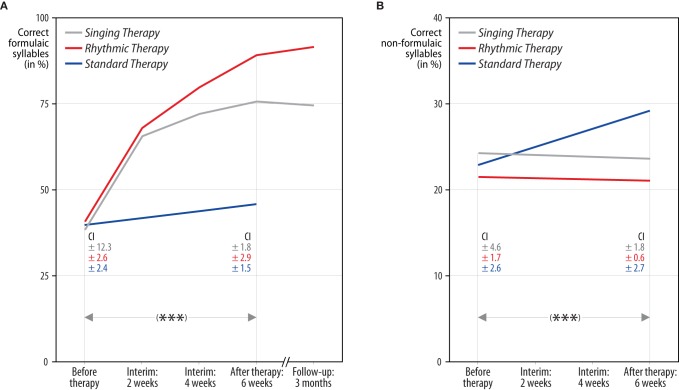
**Correctly produced formulaic and non-formulaic lyric syllables in each treatment group.** The results yielded a significant interaction of time, treatment group, and lyric type (^***^p < 0.001). Panel **(A)**: both singing and rhythmic therapy patients improved their production of formulaic phrases (“Hello, everything alright? Everything's fine…”). This progress occurred at an early stage of both therapies and was stable over time. Conversely, patients receiving standard speech therapy made less progress in the production of formulaic phrases. Panel **(B)**: standard therapy patients improved their production of non-formulaic speech (“Bright forest, there at the boat, thin like oak…”), in contrast to singing and rhythmic therapy patients, who did not. Hence, only standard therapy patients showed a training transfer to the production of unknown phrases. All values are averaged across modality (sung, spoken) and adjusted for baseline differences between treatment groups, as revealed by an analysis of covariance. The numbers below represent confidence intervals (CI) for each measurement before and after singing therapy (gray), rhythmic therapy (red), and standard therapy (blue). Confidence intervals are corrected for between-subject variance (Loftus and Masson, 1994).

A repeated measures ANCOVA, based on articulatory quality, revealed a significant interaction of time, treatment group, and lyrics [*F*_(2, 11)_ = 49.86, *p* < 0.001, partial η^2^ = 0.90]. Comparing the means before and after each treatment, strong increases in the production of formulaic lyrics were found for patients undergoing singing therapy (mean increase [*M*] and confidence interval [CI]: *M* = 36.47, 95% CI [28.24, 44.70]), and rhythmic therapy (*M* = 50.40, 95% CI [42.17, 58.63]). These effects proved to be stable over a period of 3 months after the end of singing and rhythmic therapy (*M* = −0.74, 95% CI [−3.84, 2.35]; *M* = 2.76, 95% CI [−2.82, 8.34]). Standard therapy patients showed a smaller increase in the production of formulaic lyrics (*M* = 4.98, 95% CI [−3.25, 13.21]). For the production of non-formulaic lyrics, the results yielded the reverse pattern: only standard therapy patients improved (*M* = 6.21, 95% CI [3.96, 8.47]), which was not the case with singing and rhythmic therapy patients (*M* = −0.36, 95% CI [−2.62, 1.90]; *M* = −0.50, 95% CI [−2.76, 1.76]). No significant interactions were found for modality and treatment group [*F*_(2, 11)_ = 1.44, n.s.]. Moreover, the data did not reveal a significant interaction between time and baseline scores [*F*_(1, 11)_ = 1.24, n.s.]. Estimated marginal means of the ANCOVA, averaged across modality and adjusted for baseline differences between treatment groups, are shown in Figure 2. Raw means are given in Tables 4 and 5.

**Table 4:** **Formulaic lyrics**.

**Time**	**Singing**	**Rhythmic**	**Standard**
	**therapy**	**therapy**	**therapy**
Before therapy: sung	43 (±10.4)	27 (±11.1)	42 (±2.5)
Before therapy: spoken	47 (±12.3)	28 (±2.6)	49 (±2.4)
Interim, 2 weeks: sung	71 (±7.4)	56 (±4.2)	^*^
Interim, 2 weeks: spoken	72 (±3.1)	57 (±3.8)	^*^
Interim, 4 weeks: sung	78 (±5.1)	66 (±7.0)	^*^
Interim, 4 weeks: spoken	78 (±1.4)	71 (±6.8)	^*^
After therapy, 6 weeks: sung	82 (±3.4)	77 (±1.4)	48 (±1.8)
After therapy, 6 weeks: spoken	82 (±1.8)	79 (±2.9)	53 (±1.5)
Follow-up, 3 months: sung	82 (±3.4)	78 (±4.5)	^*^
Follow-up, 3 months: spoken	81 (±3.5)	82 (±6.9)	^*^

Values represent correct syllables (in %) of formulaic lyrics at different stages of each treatment. Values in brackets display confidence intervals corrected for between-subject variance (Loftus and Masson, 1994)

* No interim or follow-up measurements were conducted in this group (see “Measurements”).

**Table 5:** **Non-formulaic lyrics**.

**Time**	**Singing**	**Rhythmic**	**Standard**
	**therapy**	**therapy**	**therapy**
Before therapy: sung	27 (±3.4)	11 (±0.6)	23 (±4.3)
Before therapy: spoken	32 (±4.6)	13 (±1.7)	32 (±2.6)
After therapy, 6 weeks: sung	27 (±2.8)	11 (±1.9)	31 (±1.5)
After therapy, 6 weeks: spoken	31 (±1.8)	12 (±0.6)	37 (±2.7)

Values represent correct syllables (in %) of non-formulaic lyrics before and after 6 weeks of treatment. Values in brackets display confidence intervals corrected for between-subject variance (Loftus and Masson, 1994)

To further explore the current findings, two *post-hoc* analyses were performed, each based on the production of formulaic lyrics in singing and rhythmic therapy patients after 6 weeks of treatment. *First*, the analyses explored whether singing and rhythmic therapy may have altered the phonatory quality of the patients' voice. More precisely, it was assessed whether singing and rhythmic therapy affected the rate of continuous phonation in the patients' sung and spoken utterances. The rate of continuous phonation was denoted as the percentage of voiced articulation during each sung and spoken syllable, as measured with *Praat*. Syllable omissions were discarded from the analyses. The results revealed a higher average rate of continuous phonation during singing (79%) compared to rhythmic speech (68%; Wilcoxon signed-rank test: *z* = 2.78, *p* = 0.005). This finding was independent of whether patients had previously undergone singing or rhythmic therapy (Mann–Whitney *U* test for sung and spoken performances, *z* ≤ 0.63, always n.s.). The second analysis investigated whether singing therapy has affected prosody or, more technically, the variance of vocal fundamental frequency. Fundamental frequency variances were computed based on frequency listings with 10 data points per second, as indicated by *Praat*. The results revealed higher fundamental frequency variances during rhythmic speech (mean variance: 1531 Hz) as compared to singing [725 Hz; *F*_(9, 9)_ = 9.00, *p* = 0.002]. This finding did not depend on whether patients had previously undergone singing or rhythmic therapy (Mann–Whitney *U* test for sung and spoken performances, *z* ≤ 1.04, always n.s.).

## Discussion

The current longitudinal experiment investigated the relative effects of melody and rhythm on the recovery of formulaic and non-formulaic speech. Fifteen patients with chronic non-fluent aphasia underwent either singing therapy, rhythmic therapy, or standard speech therapy. The experiment controlled for phonatory quality, vocal frequency variability, pitch accuracy, syllable duration, phonetic complexity and other influences, such as the acoustic setting and learning effects induced by the testing itself. The longitudinal results suggest that singing and rhythmic speech may be similarly effective in the treatment of non-fluent aphasia. Both singing and rhythmic therapy patients made good progress in the production of common, *formulaic* phrases. This progress occurred at an early stage of both therapies and was stable over time. Independent of whether patients had received singing or rhythmic therapy, they were able to easily switch between singing and rhythmic speech at any time. Conversely, patients receiving standard therapy made less progress in the production of formulaic phrases. They did, however, improve their production of *non-formulaic* speech, in contrast to singing and rhythmic therapy patients, who did not. In other words, only standard therapy patients showed a training transfer to the production of unknown phrases. Overall, treatment and lyric type accounted for about 90% of the variance related to speech recovery in the data reported here.

The current results suggest that singing may not benefit speech recovery over and above rhythmic speech. One may nevertheless argue that singing could have a positive long-term effect on *phonatory quality*, for example by enhancing respiratory activity. Such an effect seems all the more possible, as the choral element of singing is used to increase the rate of continuous phonation in voice therapy, especially in stuttering patients (Thyme-Frøkjær and Frøkjær-Jensen, 2011). Indeed, the present data reveal a slightly increased rate of continuous phonation during singing as compared to rhythmic speech (for similar evidence in stuttering patients, see Colcord and Adams, 1979). However, this result was independent of whether patients had previously undergone singing or rhythmic therapy. That is, the current findings do *not* support the idea that singing may have a long-term effect on phonatory quality in aphasic patients. Rather, the results indicate that singing increases the rate of continuous phonation without any prior training. Although this effect appears to be relatively small, it nonetheless suggests that singing may provide a promising tool in voice therapy. This finding may be an interesting by-product of the present experiment.

Both singing and prosody depend on vocal frequency, albeit in different ways. One may therefore imagine that singing has a long-term effect on *prosody*, such as by engaging a frontolateral network in the right hemisphere (Meyer et al., 2004). Yet, the current data do not support this claim. Variability in vocal fundamental frequency did not depend on whether patients had previously undergone singing or rhythmic therapy. That is, treatment type did not affect the amount of prosody in the patients' spoken utterances. Hence, it seems rather unlikely that singing has a long-term effect on the amount of prosody in non-fluent aphasic patients. Somewhat surprisingly, both singing and rhythmic therapy patients showed increased vocal frequency variability during *rhythmic speech* as compared to when singing. Upon closer consideration, this finding makes sense: the melody used in the present experiment did not exceed the range of a fifth, whereas natural prosody often does (Hammerschmidt and Jürgens, 2007). In other words, we tend to vary more in vocal frequency when we speak than when we sing—at least when comparing natural prosody to simple melodies.

As with any clinical trial study, a number of caveats deserve closer attention. The first critical point concerns sample size. One may argue that the sample size in the current experiment was too small to deliver universally valid results. In fact, large sample trials with aphasic patients are certainly more than desirable. Unfortunately, this claim is difficult to reconcile with the constraints of clinical practice. Homogeneous samples of motivated patients with specific lesions and speech production disorders are difficult to find—even in multicenter studies over the course of several years, as is the case in the present work. Although the current sample included only 15 patients, the sample was comparably homogeneous in terms of lesion site and symptom variability across the different treatment groups. In contrast, previous longitudinal studies on related topics have been based mainly on single patient cases. Furthermore, all of the results reported here are statistically significant.

It should be noted that the current experiment did not include a control treatment for rhythmic therapy. Such a control treatment could be focused on the training of formulaic phrases, but in a non-rhythmic or rhythmically reduced way. Hence, the present results do not warrant any final conclusions with regard to clinical efficacy of rhythm as such. However, several longitudinal studies that did include non-rhythmic control conditions provide strong evidence for the efficacy of rhythmic pacing in aphasic and apractic patients (Rubow et al., 1982; Pilon et al., 1998; Brendel and Ziegler, 2008). Although the studies differ in the type of treatment and control condition, the results clearly indicate an articulatory benefit from rhythmic pacing. Moreover, a clinical effect from rhythmic pacing is consistent with current theories of auditory-motor learning (Thaut et al., 1999; Sakai et al., 2004). Acting as a pacemaker, rhythm may help to overcome problems initiating and segmenting words at the syllable level (Cutler and Norris, 1988). This may be especially important for patients with apraxia of speech, who typically have problems in speech-motor planning, including syllabic segmentation. That is, the crucial role of rhythmic pacing in speech recovery may be substantively dependent upon the fact that non-fluent aphasic patients commonly show apractic symptoms, as is the case with the present sample.

One may argue that the current experiment should have included an additional control treatment that focuses on melodic or rhythmic training of non-formulaic, propositional speech. However, there is a fundamental difference between training of formulaic speech and propositional utterances. Formulaic speech covers a typical communicative repertoire of phrases that can be repetitively trained. Propositional utterances are by definition newly created expressions that cannot be trained in a similarly repetitive manner. This may explain why singing and rhythmic therapy patients greatly increased their production of formulaic phrases, while standard therapy patients made rather little progress in the production of non-formulaic speech. In fact, a number of experimental problems occur if propositional utterances are to be trained repetitively in a melodic or rhythmic fashion. The problems involve: stimulus control; training intensity; and the fact that spontaneous, propositional utterances almost always include formulaic strings (e.g., “I am” or “I have”). The question of whether or how to combine melody, rhythm, and training of propositional speech may therefore be more properly addressed in a research project of its own.

A look at the performance levels before treatment in the present study indicates lower averages for rhythmic therapy patients. Different baselines before treatment are critical, as they may limit the validity of comparisons between the groups. A closer look at the data reveals two important characteristics of the current sample. *First*, individual performances before treatment varied considerably in singing and rhythmic therapy patients. For this reason, baseline scores were included in the analysis as a covariate in order to control for pre-treatment differences between subjects (Overall and Doyle, 1994; Van Breukelen, 2006). *Second*, lower pre-treatment averages in the rhythmic therapy group are mainly due to the poor performance of one patient (patient PH). If this patient is discarded from the analyses, the baseline differences between singing and rhythmic therapy patients disappear almost completely.

It is clear that interviews with the patients' relatives can only offer limited insight regarding the extent to which formulaic phrases are employed in real life. Nonetheless, the present interviews yielded some interesting results. For example, patients were using a fixed number of formulaic phrases successfully in communicative contexts. In a way, patients were establishing their own individual formulaic repertoire that varied substantially from patient to patient. Furthermore, individual patients showed different patterns in how they depended on *external cues* to initiate phrase production. External cues involved: rhythmic beats of various kind; onset syllables; and small cards labeled with phrases. Two patients (patients LS and PH) showed difficulties in self-initiating phrase production throughout the treatment. Other patients (patients IK, OK, PL, PR, AS, DO, GB, and HG) became gradually independent of external cues, applying a number of self-pacing strategies—such as silent upbeat counting. In sum, the interviews suggest a considerable progress in most patients, notably in a short time.

**Figure 3:**
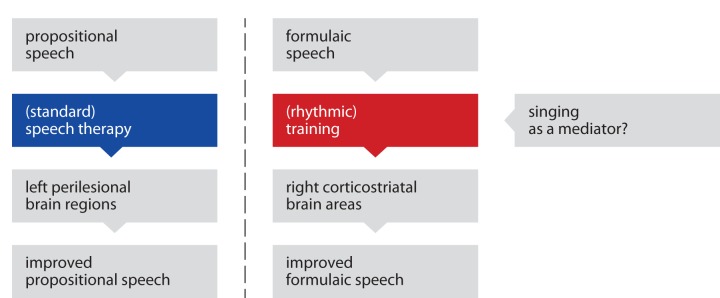
**Two-path model of speech recovery.** The recovery of propositional and formulaic speech may rely on different neural pathways. Propositional speech may be improved through standard speech therapy, engaging primarily left perilesional brain regions. Formulaic speech may be rhythmically trained, engaging right corticostriatal brain areas. At least theoretically, singing could mediate this training process.

The current results are consistent with the idea that propositional and formulaic speech rely on different neural pathways (Van Lancker Sidtis, 2004). One may therefore propose that therapy of non-fluent aphasia should focus on *both* propositional and formulaic speech, as illustrated in the model shown in Figure 3. Propositional speech may be improved through standard speech therapy, engaging left perilesional brain regions (Cao et al., 1999; Warburton et al., 1999; Kessler et al., 2000; Rosen et al., 2000; Zahn et al., 2004; Meinzer et al., 2008). Formulaic speech may be rhythmically trained, engaging right corticostriatal areas (Speedie et al., 1993; Van Lancker Sidtis et al., 2003; Van Lancker Sidtis and Postman, 2006; Sidtis et al., 2009). At least theoretically, singing could, nonetheless, mediate this training process, perhaps by motivating patients or—neurophysiologically—by triggering the reward system (Menon and Levitin, 2005; Emanuele et al., 2009). However, it should be noted that motivating or rewarding effects could just as well arise from rhythmic features (Kokal et al., 2011; Rothermich et al., 2012). In other words, future research may need to address a number of *non-articulatory* aspects of singing and rhythmic speech in the treatment of non-fluent aphasia.

It is likely that the model presented here oversimplifies a number of concurrent processes in the brain, about which little is known so far. This is especially true as the current experiment is based on the conclusions of previous neuroimaging studies, but does not include neuroimaging itself. For example, it remains unclear *to what degree* propositional utterances and formulaic speech rely on different neural mechanisms. The two-path model of speech recovery presented here serves two purposes. *First*, the model aims to critically appraise related findings from the last few decades and to integrate them with the findings from the current experiment in a meaningful way. *Second*, the model accounts for both propositional and formulaic language and may thus provide a useful heuristic in speech therapy.

### Conflict of interest statement

The authors declare that the research was conducted in the absence of any commercial or financial relationships that could be construed as a potential conflict of interest.
